# Influence of Polymer Nature on Electroadhesion

**DOI:** 10.3390/polym16233344

**Published:** 2024-11-28

**Authors:** Konstantin I. Sharov, Valentina Yu. Stepanenko, Uliana V. Nikulova, Aleksey V. Shapagin

**Affiliations:** Frumkin Institute of Physical Chemistry and Electrochemistry Russian Academy of Sciences (IPCE RAS), 31, bld. 4 Leninsky Prospect, 119071 Moscow, Russia; deathknight_91@mail.ru (K.I.S.); ulianan@rambler.ru (U.V.N.)

**Keywords:** electroadhesive, permittivity, electric field, dipole, electroadhesive force, dipole orientation, epoxy, polyurethane, polyester

## Abstract

Electroadhesive systems are promising for creating delicate robotic manipulators operating both in the natural environment and in space conditions. Using thermosetting epoxy resin, polyurethane and polyester resin as examples, the influence of the polymers’ natures, potential differences and current strengths on electroadhesive interactions in polymer–polymer systems was studied. The investigations were carried out by recording the force of normal separation of substrates from electroadhesives using contact and contactless methods at various electrical parameters of the systems and their components. A correlation was established between the relative permittivity and the electroadhesive force. The relaxation nature of the electroadhesion phenomenon after removing the electrical voltage was revealed. The influence of the potential difference and current strength on the effect of electroadhesion for polymer substrates of various natures was established. The obtained dependencies describe the main regularities of electroadhesive interactions necessary for creating promising electroadhesive materials.

## 1. Introduction

**Figure 1:**
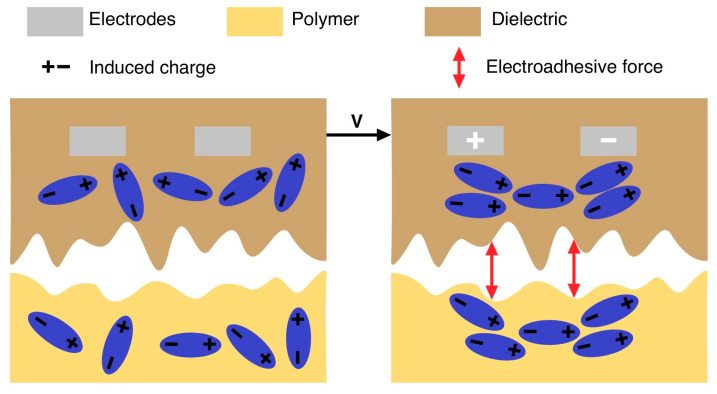
Mechanism of electroadhesion in polymer materials.

Electroadhesion (EA) is a complex dynamic effect of electrostatic attraction between an electroadhesive and an object, depending on a huge number of factors (~33 variables) [1]. The mechanism of the appearance of the electroadhesion effect in dielectric polymer materials is shown in Figure 1.

The magnitude of the electroadhesive forces in dielectric materials is determined by total polarization and is calculated using Equation (1):

(1) $$F_{EA}=P_{T}E\;P_{T}=P_{e}+P_{i}+P_{o}+P_{s}+P_{f}$$

where E is the electric field strength, $P_{e}$ is the electronic polarization, $P_{i}$ is the ionic polarization, $P_{o}$ is the orientational (dipole) polarization, $P_{s}$ is the spontaneous polarization and $P_{f}$ is the interfacial polarization.

Despite the large number of possible polarizations, it is assumed that the orientational (dipole) polarization ($P_{o}$) has the greatest influence on the forces of electroadhesion in polymer materials, and therefore the other terms of the equation can be neglected [1].

Currently, the phenomenon of electroadhesion has a wide range of applications, which is well described in the review [1]. In particular, the phenomenon of electroadhesion is used in various industries in the electroadhesive grips of manipulators and in robotics as part of crawling and climbing robots, and also has good prospects for use in the space industry in devices for docking space modules and catching space debris. Electroadhesion has a number of advantages over other controlled adhesives, such as suction adhesives, electromagnetic and dry adhesives. The advantage of electroadhesives is their increased technological effectiveness, since the electroadhesion effect appears in many materials—conductive, semiconductive or dielectric ones—and on any surface—smooth or rough [2]. The electroadhesive effect also functions well in a vacuum, which means that electroadhesives can be used in space conditions. Another advantage of electroadhesives is the delicacy of manipulations with materials, since there is no mechanical contact. This is extremely important when working with fragile materials, so electroadhesive grippers are widely used in the semiconductor industry for the manufacture of micro-electromechanical systems [3,4]. Another advantage of electroadhesives is the simplicity of the system, since all operations are electrically controlled. Accordingly, there is no need for various pumps and motors, which significantly reduces the weight of the construction. Another undeniable advantage of electroadhesives is their low energy consumption in the range from microwatts to milliwatts, since extremely small currents in the range from microamperes to milliamperes pass through the electroadhesive. However, with all their advantages, electroadhesives have one significant drawback—a relatively low adhesive force compared to adhesives that operate on other adhesive mechanisms [1,5,6].

Currently, there are a large number of works on simulation electroadhesive forces that study the influence of the electrode form factor and surface roughness on electroadhesive forces [2,3,6,7,8,9,10,11,12,13]. There are many applied works [4,5,14,15,16,17,18,19,20,21,22,23,24,25,26]. The use of electroadhesion in touch screens has been widely considered [18,21,25,26] and the combination of two mechanisms—dry adhesion and electroadhesion [14]—has been described in some detail, as has the simplification of the technology for manufacturing electroadhesives [17]. There are some published works devoted to the use of various materials for electroadhesives [15,19,23] and the control of the electroadhesion effect [16,24]. However, there are quite a few fundamental studies on the influence of the nature and structure of the material on the effect of electroadhesion.

This work is focused on the search for the main dependencies of the electroadhesion effect on polymer substrates of various natures with the aim of applying the obtained knowledge in further studies of structure formation in multicomponent reactive systems [27,28] and the influence of the energy characteristics of polymers on interphase interactions under the influence of an electric field.

Thus, the aim of the work was a comprehensive study of electroadhesive polymer–polymer systems based on materials of different natures in wide ranges of high-voltage values of electric voltage and current, as well as a study of the kinetics of the electroadhesive contact formation and the relaxation of electroadhesive interactions in the presence and absence of an electric field, respectively.

## 2. Materials and Methods

### 2.1. Materials

The studied objects were electroadhesive systems: electroadhesive substrate.

**Figure 2:**
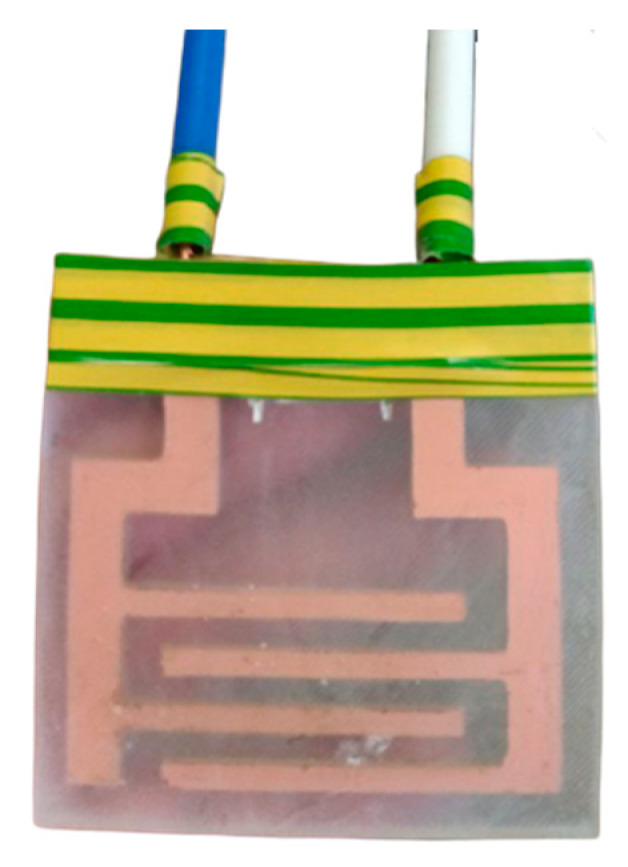
Electroadhesive based on interdigital electrodes.

**Figure 3:**
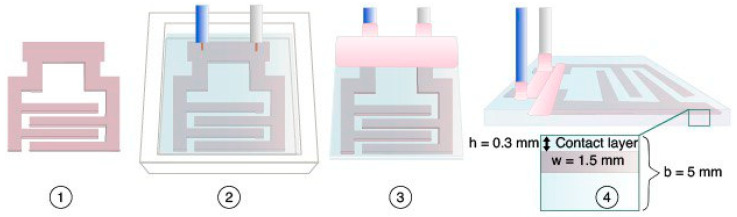
Electroadhesive manufacturing technology: h is the contact layer thickness, w is the electrode thickness, b is the total thickness. Explanations in the text.

An electroadhesive (Figure 2) is a dielectric plate with interdigital electrodes built into it, manufactured using the technology shown in Figure 3. An electrode was cut from foil-clad textolite on a CNC machine (Figure 3, step 1). The distance between the pins was 5 mm, the width of the pins was 4 mm and the thickness of the contact layer was 0.3 mm. The contacts were soldered and the electrode in a silicone mold was filled with a dielectric compound and cured (Figure 3, step 2). Polyurethane and epoxy resin were used as compounds. The connecting bridge was removed and the area of the contact pads was isolated (Figure 3, step 3).

**Figure 4:**
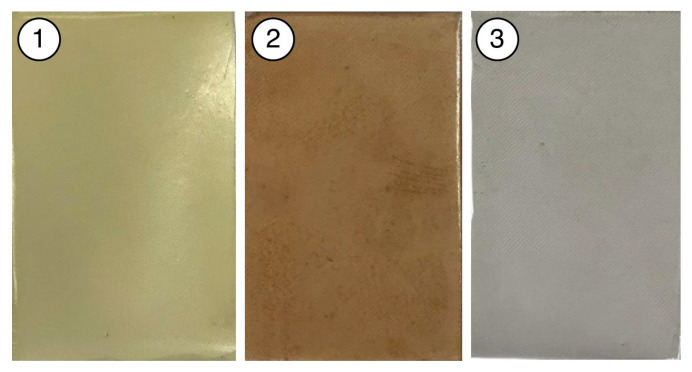
Photo images of substrates made of cured polyurethane (1), polyester (2) and epoxy (3) resins.

Polymer plates made of cured polyurethane (NOACAST 700, Kompozit stroy, Moscow, Russia), polyester (NOAPOL 740L, Kompozit stroy, Moscow, Russia) and epoxy resins (Sipo art label, Moscow, Russia) of 80 × 50 mm area with thicknesses of 3.7 mm, 3 mm, 1.8 mm and a relative permittivity of 5.5, 7.1 and 7.5, respectively, were used as substrates (Figure 4).

### 2.2. Methods

**Figure 5:**
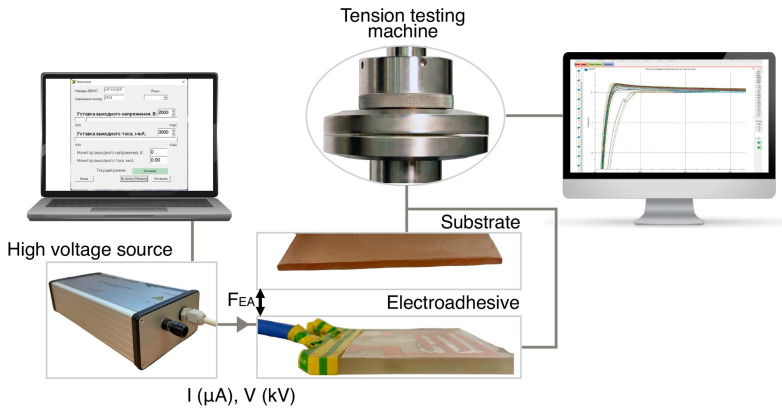
Block diagram of the electroadhesive system and experiment.

The block diagram of the electroadhesive system and additional equipment used to create and record electroadhesive forces is shown in Figure 5. A HT-15-20-P current source (Mantigora, Novosibirsk, Russia) was used to generate voltage (from 2 to 10 kV). The high voltage source supplies a specified value of current (I) and voltage (V) to the electroadhesive, resulting in a charge of the surfaces of the electroadhesive and the substrate in contact with it; the electroadhesive force (F_EA_) is generated, and a strong electrostatic attraction (electroadhesive contact) occurs between their surfaces. F_EA_ is recorded by the force sensor of the tensile testing machine at the moment of substrate removal.

**Figure 6:**
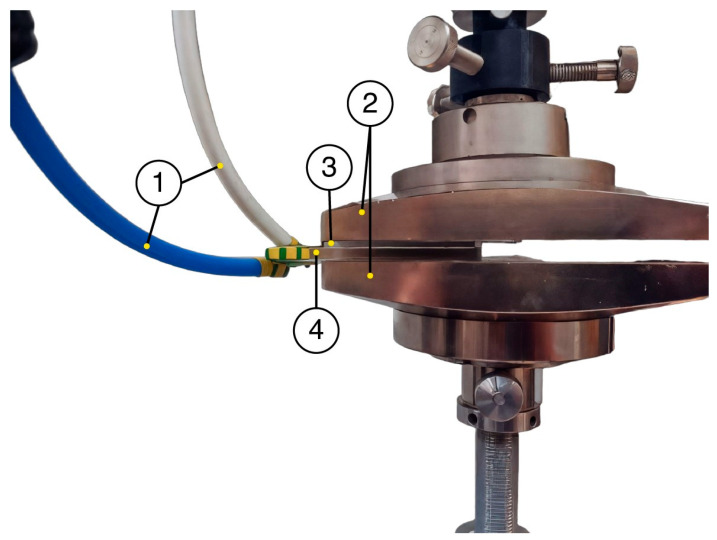
Photo image of the experimental setup, where 1 is the high voltage wires connecting the current source and the electroadhesive, 2 is the plates of the testing machine with the electroadhesive and substrate fixed to them, 3 is the substrate and 4 is the electroadhesive.

Adhesion measurements were carried out on a Z010 testing machine (Zwick/Roell, Ulm, Germany) equipped with a force sensor with a nominal load of 200 N and an accuracy class of 0.25 (Figure 6). The reproducibility of the results was assessed based on 5 measurements of electroadhesive systems at each point. The variation coefficient did not exceed 5%.

**Figure 7:**
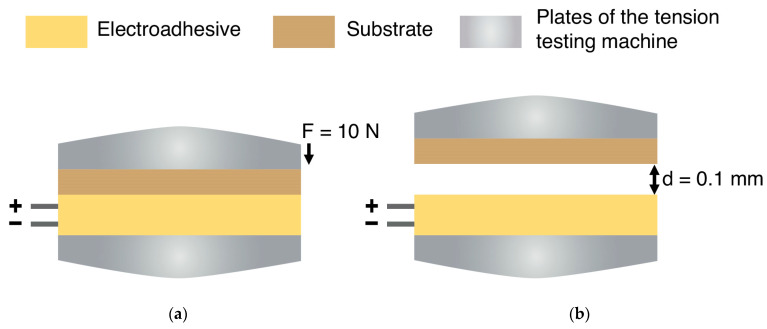
Schemes of contact (**a**) and contactless (**b**) methods.

The experiments were carried out using two methods: contact and contactless (Figure 7).

Method No. 1 was an electroadhesive scheme, according to which the electroadhesive was brought into contact with the substrate with a force of 10 N. Then, electric voltage was applied to the electroadhesive for 30 s. After that, without removing the electric voltage, a normal tear was performed at a speed of 2 mm/min. The change in force relative to the movement of the electroadhesive relative to the substrate was recorded.

In the case of contactless Method No. 2, a gap of 0.1 mm was set between the electroadhesive and the substrate. The method for achieving the gap: 1. the tensile testing machine plates with the electroadhesive and substrate fixed to them are brought into contact with a preload of 10 N; 2. using the software application of the TestXpert II testing machine, the crosshead is moved and the plates with the adhesive system components are moved apart to a specified gap of 0.1 mm. Then, with the minimum speed of the crosshead movement (to start recording the adhesive force in the quasi-static state of the electroadhesive system), the kinetics of charging the surfaces of the electroadhesive and substrate and discharging after removing the electrical voltage were determined at a given value of electrical voltage (applied for at least 30 s) at intervals of 10, 20, 30 and 60 s. Method No. 2 shows the features of the operation of the electroadhesive system with loose contact of the surfaces.

Both methods, with a step-by-step algorithm and the value of constants and ranges of variable parameters, are presented below.

Method No. 1. Contact. (Figure 7a):

(1) Bring the electroadhesive and substrate into contact with a preload of 10 N.
(2) Apply an electric voltage in the range from 2 to 9 kV.
(3) Charge the electroadhesive and plate for 30 s.
(4) Perform a normal tear-off at a speed of 2 mm/min without removing the electric voltage.
(5) Switch off the current source and discharge the electroadhesive and substrate for 120 s.

Method No. 2. Contactless. (Figure 7b):

(1) Set the gap between the electroadhesive and the substrate to 0.1 mm.
(2) Turn on the movement of the crosshead with a minimum breakaway speed of 0.000001 mm/hour.
(3) Alternately apply and remove electrical voltage in the range from 2 to 9 kV with discharge intervals of 10, 20, 30 and 60 s (the charging of the surfaces of the electroadhesive and substrate takes 30 s).

## 3. Results and Discussion

**Figure 8:**
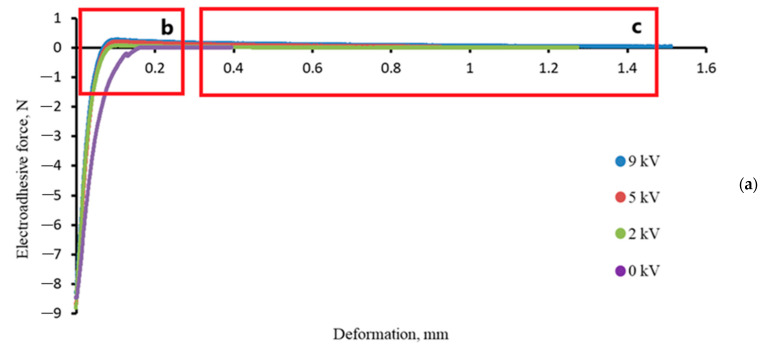

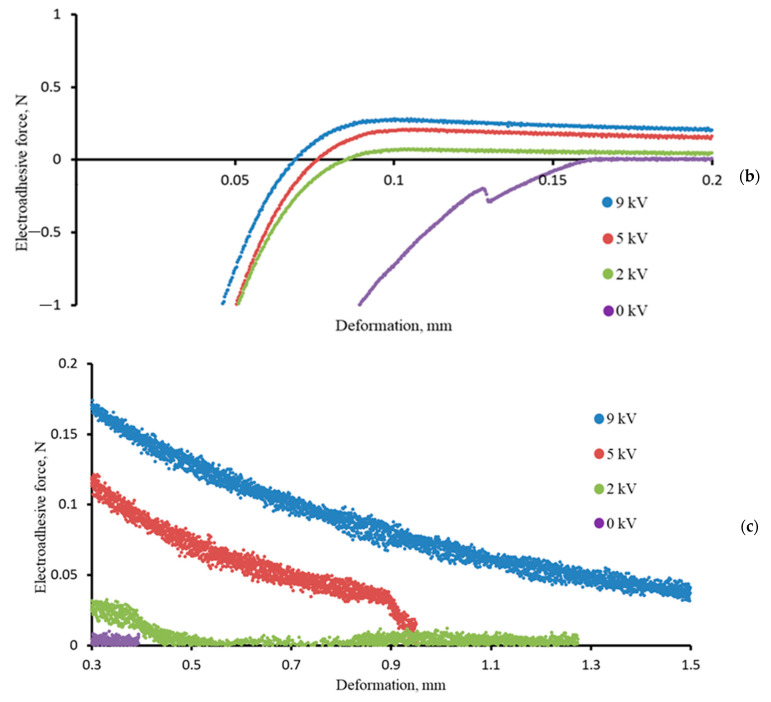
(**a**) Initial adhesion graphs of the epoxy electroadhesive-polyurethane substrate systems obtained using Method 1; (**b**) enlarged to scale initial fragment of the initial adhesion graphs; (**c**) enlarged to scale fragment of the initial adhesion graphs with an emphasis on the cessation of electroadhesive interactions F_EA_ < 0.05.

Figure 8 shows adhesion profiles obtained using Method 1 in force-deformation coordinates. It is evident that as the mechanical stress specified by the preload is removed, electroadhesive forces are realized in the system. The electroadhesive substrate system, in which electroadhesive interactions were not excited after the application of preload, was used as a zero comparison object. The difference in the forces expended on the destruction of systems with and without the application of electrical voltage was established.

**Figure 9:**
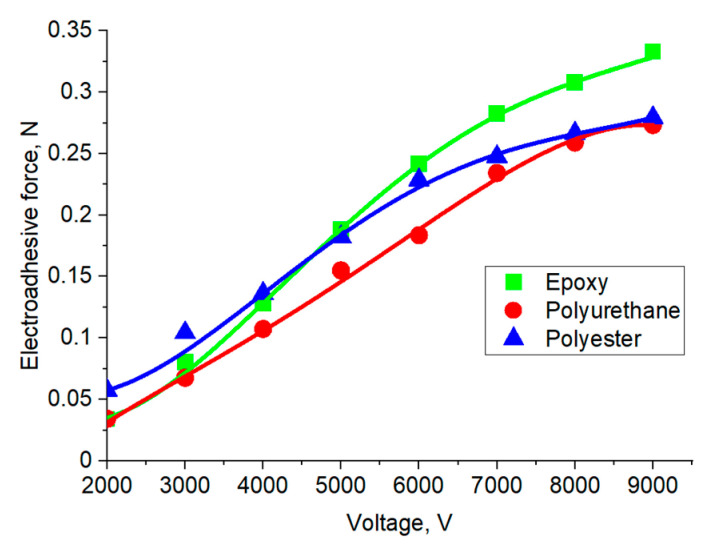
Dependence of electroadhesive forces on electrical voltage for polyurethane-based electroadhesive (contact method) and various substrates.

**Figure 10:**
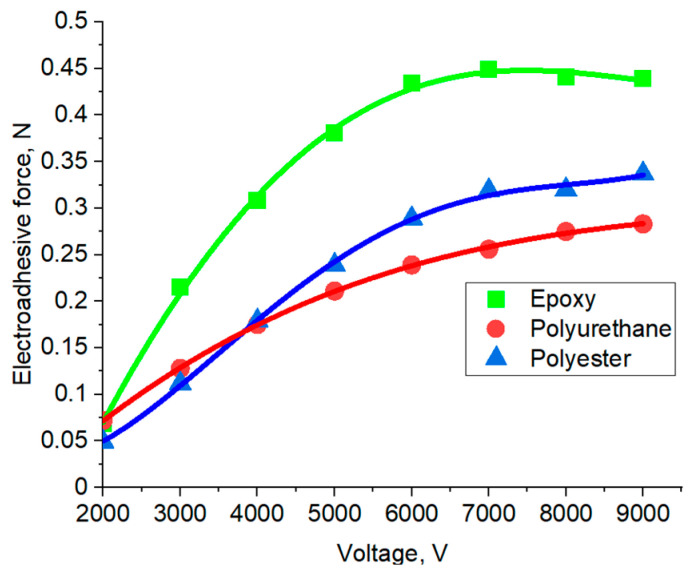
Dependence of electroadhesive forces on electrical voltage for an epoxy resin-based electroadhesive (contact method) and various substrates.

Based on the adhesion graphs obtained using the contact method, the dependences of the forces arising in the electroadhesive systems on the applied electrical voltage were constructed (Figure 9 and Figure 10). The value of the electroadhesive force was determined by the maximum on the curve. It was shown that with an increase in electrical voltage, the force of destruction of electroadhesive systems increases, which is due to the increase in the magnitude of dipole polarization in the volume of materials.

At the initial stage, the influence of the electroadhesive material in relation to the substrate material on the forces of interface interactions occurring in the electroadhesive system at different values of electric voltage was determined. It was found that the greatest forces of destruction of the electroadhesive system are achieved when using the electroadhesive and substrate made of materials with the maximum values of the dielectric constant. That is, as noted above, the ability of the dielectric material of the electroadhesive to polarize electric dipoles, which is important. Thus, the cured epoxy resin, in which the electrodes of the electroadhesive were poured, characterized by a dielectric constant of 7.5, contributed to an increase in the resistance to the destruction of the adhesive joint when using an epoxy substrate. It is important that in electroadhesive systems in which the electrodes were poured with a polyurethane compound with a dielectric constant of 5.5, the influence of the substrate on the force of destruction of the systems was practically not affected. That is, to achieve the maximum effect, the most important is the material of the electroadhesive, since it is in direct contact with the electrodes, which leads to a better orientation of the dipoles of the electroadhesive compared to the substrate. The influence of the materials of the electroadhesive and the substrate is clearly illustrated by a comparative analysis of the dependencies presented in Figure 9 and Figure 10.

Thus, using an electroadhesive with a better polarization of dipoles in the volume leads to better dipole–dipole interaction with other materials, with large values of permittivity. In this regard, further in the work, we used an electroadhesive based on cured epoxy resin.

**Figure 11:**
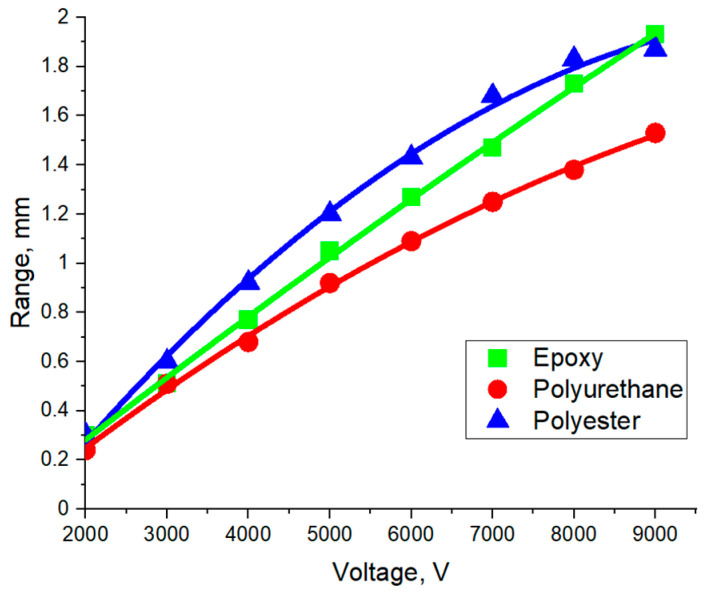
The influence of electrical voltage on the maximum range of excitation of electroadhesion in systems based on epoxy electroadhesive and substrates of different nature.

The adhesion graphs obtained using contact Method 1 were also used to study the range of action of electroadhesive forces depending on the electric voltage applied to the electroadhesive (Figure 11). The parameter characterizing the range of action of dipole–dipole interactions was the distance between the electroadhesive and the substrate at which the recorded value of electroadhesive forces approached zero, or more precisely became less than 0.05 N.

It is shown that when a gap occurred, the electroadhesive interactions between the cured epoxy electroadhesive and the substrate are weakened, which is due to the difficulty of polarizing the electric dipoles in the substrate. With an increase in the voltage applied to the electroadhesive, the range of recording the electroadhesive effect increases and the influence of the nature of the substrate on this characteristic increases. Materials with higher values in the dielectric constant retain the ability to generate dipole–dipole interactions at greater distances.

**Figure 12:**
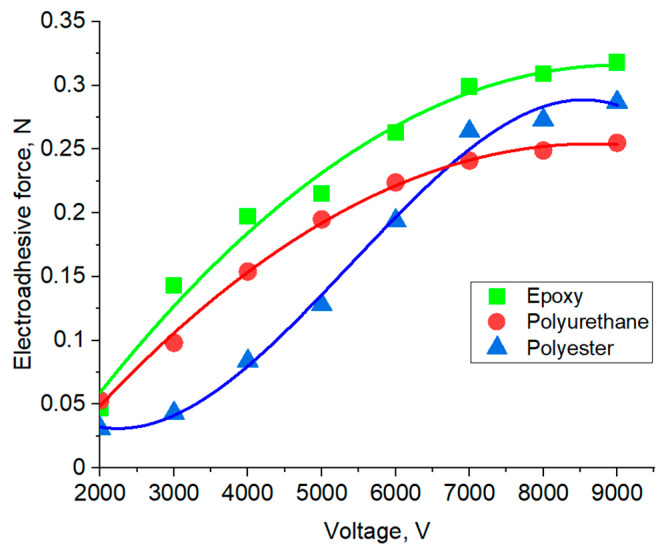
Dependence of electroadhesive forces on electrical voltage for electroadhesive based on cured epoxy resin (contactless method) and substrates of different nature.

The contactless method is used to solve the applied problems of using electroadhesion in the absence of close contact between materials. It is evident that when voltage is applied to the electroadhesive, the increase in dipole–dipole interactions occurs faster in electroadhesive pairs formed by the electroadhesive and the substrate based on materials with higher dielectric constant values. Figure 12 summarizes the data obtained using the contactless method in the electrical voltage—force coordinates. Note that the 0.1 mm gap between the plates of the electroadhesive and the substrate did not lead to significant differences in the values of the electroadhesive forces recorded compared to the contact method. This is due to the fact that the decrease in the electroadhesive forces recorded using Method 1 begins at a distance of about 0.1 mm between the electroadhesive and the substrate. It is important that all dependencies reach a plateau when applying an electrical voltage of about 7000 V. This fact indicates that the maximum orientation in the electroadhesive system is achieved at 7000 V. A further increase in the potential difference does not lead to an increase in electroadhesive interactions.

**Figure 13:**
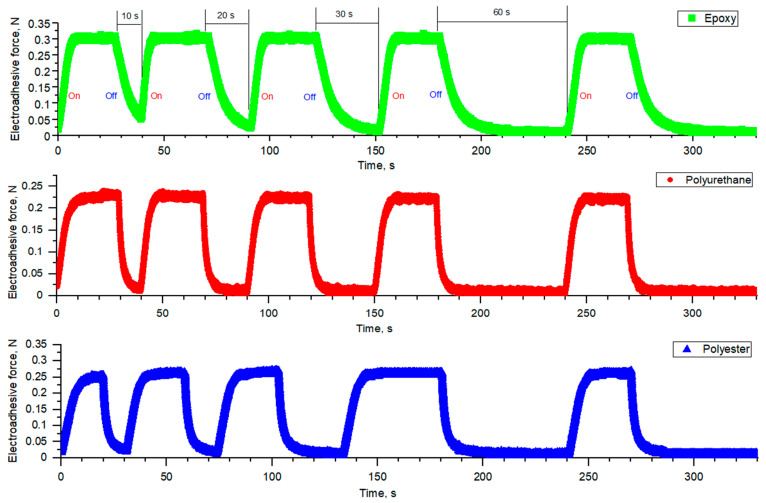
Charge–discharge diagrams at 7000 V of electroadhesive systems based on epoxy electroadhesive and substrates of different nature.

The kinetics of electric dipole orientation with subsequent relaxation when voltage is switched off was studied using a contactless method. Figure 13 shows charge–discharge diagrams of electroadhesive systems for substrates of different nature. The electroadhesive system was charged for 30 s, while discharge was carried out at different intervals: 10, 20, 30 and 60 s.

**Figure 14:**
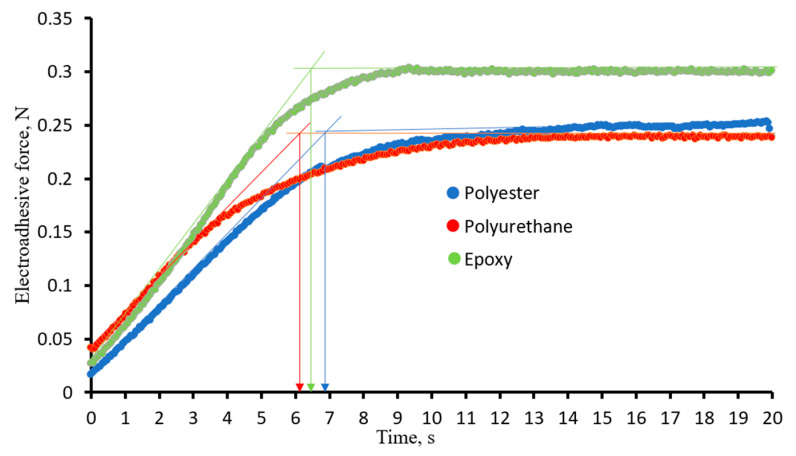
Kinetic dependences of the orientation of electric dipoles in an electroadhesive system based on epoxy electroadhesive and substrates of different nature at 7000 V.

It has been established that the dielectric constant of the substrate material affects both the kinetics of the orientation of electric dipoles and the loss of their order. Note that the higher the characteristics of the dielectric constant of the materials in the electroadhesive pair, the slower the misorientation of electric dipoles in the adhesive system. The nature of the materials has little effect on the rate of orientation of dipoles under the action of an electric field (Figure 14). The time to reach the maximum values of the dipole–dipole interaction force for the studied materials is 6–7 s (indicated by arrows in Figure 14).

**Figure 15:**
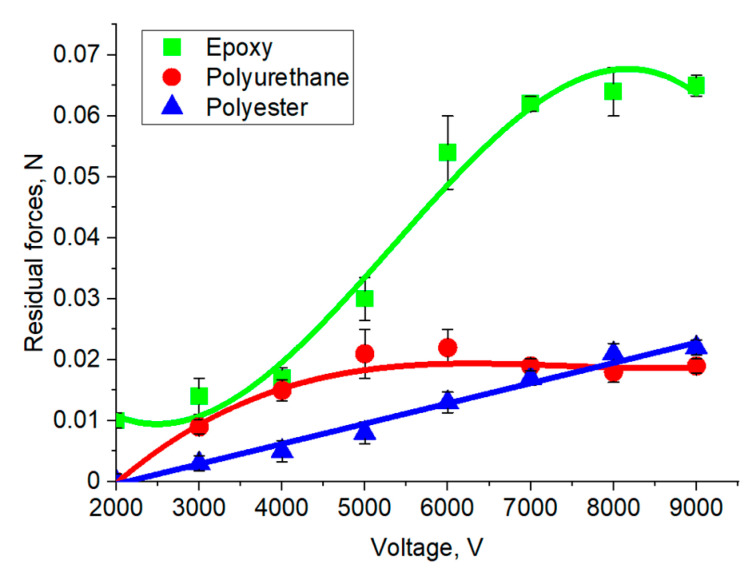
Residual electroadhesive forces 10 s after switching off the voltage at different electrical voltages for forming an electroadhesive system.

The difference in the loss of dipole ordering for substrates of different natures is clearly shown in the dependence of the electrical voltage residual forces during the relaxation of the electroadhesive effect 10 s after the voltage is removed (Figure 15). It is evident that for substrates with low values of dielectric constant, residual electroadhesive interactions are insignificant (less than 0.05 N) after 10 s. For an electroadhesive pair made of epoxy resin, with increasing voltage, the residual electroadhesive force reaches a plateau at a potential difference of 7000 V. This confirms the above conclusion about achieving the maximum orientation of dipoles in the electroadhesive system starting from 7000 V. An increase in the time of application of electrical voltage to electroadhesive systems and the relaxation time after its switching off does not affect the kinetics of the subsequent charge–discharge processes of the studied systems.

**Figure 16:**
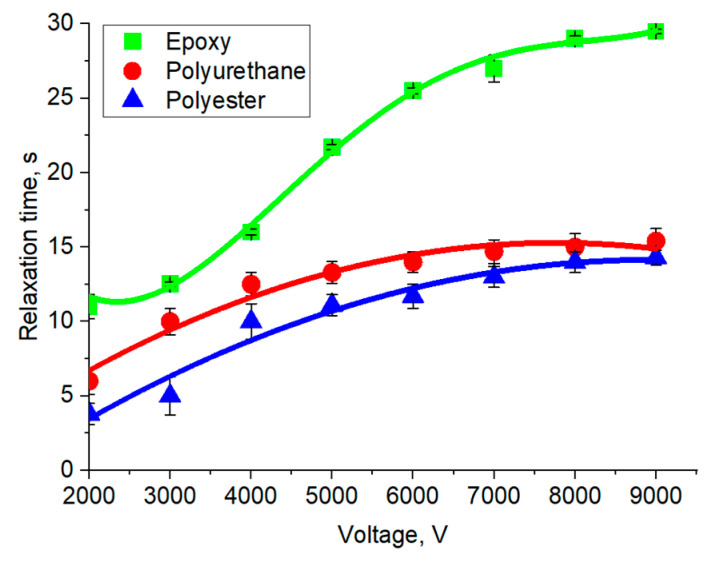
Relaxation time of electroadhesive forces depending on the applied electrical voltage.

Figure 16 shows the dependences of the relaxation times of dipole–dipole interactions on the electric voltage of the adhesive contact formation for substrates of different natures of the electroadhesive system based on the epoxy electroadhesive. Regardless of the nature of the substrate, the relaxation time of electroadhesive interactions reaches a plateau starting at 7000 V. This fact once again confirms that, regardless of the nature of the studied materials, the application of a potential difference of 7000 V to the electroadhesive system is sufficient to complete the processes of orientation of electric dipoles, ensuring the maximum electroadhesive interactions for a particular system. The maximum relaxation times of electroadhesive interactions are observed in systems with the highest values of dielectric constant. In this study, such a pair was an electroadhesive and a substrate made of epoxy resin. Thus, this system has a better ability to retain charge over time, which may be a problem for using an electroadhesive as a manipulator that must perform multiple adhesion–deadhesion cycles in a fixed time period. When using materials with lower dielectric constant values (polyurethane and polyester), residual electroadhesive forces are 2.5 times lower compared to epoxy resin, which makes such materials more relevant in devices that require a high rate of adhesion–deadhesion cycles.

**Figure 17:**
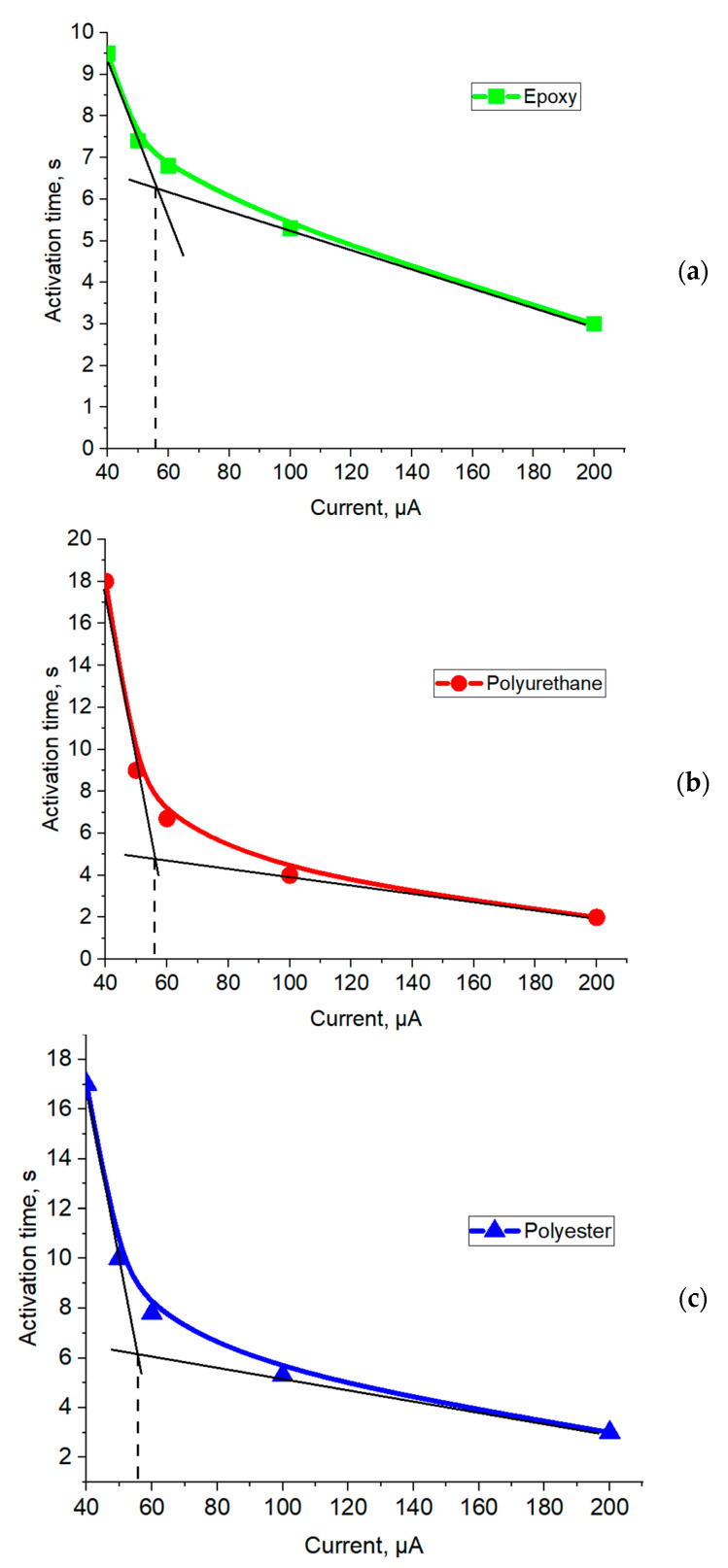
Effect of current strength on the activation time of electroadhesion in a system based on epoxy electroadhesive and substrate (**a**); on a polyurethane plate (**b**); on a polyester plate (**c**).

At the final stage, the effect of current strength on the activation time of electroadhesion (Figure 17), i.e., the time to achieve maximum electroadhesive forces, was investigated. It is evident that the activation time of electroadhesion is inversely proportional to the strength of the applied current. With increasing current strength, a greater number of electrons pass through the electroadhesive per unit of time, which leads to a faster orientation of dipoles on the surface and in the volume of the substrate. In other words, the higher the current strength, the shorter the activation time of electroadhesion.

The dependencies shown in Figure 17 are described by two lines with different slope angles. Their intersection allows us to determine the optimal current strength that allows us to achieve the best activation time of electroadhesion. That is, a further significant increase in currents leads to an insignificant decrease in the activation time of the electroadhesive contact. Note that the higher the dielectric constant of the materials of the electroadhesive systems, the more linear the function describes the dependence in the time current strength coordinates, mainly due to the decrease in the activation time of electroadhesion at low currents. It was also found that, regardless of the nature of the studied substrates, the optimal activation time of electroadhesion is achieved at a current strength of about 55 μA (shown by the dotted line in Figure 17).

## 4. Conclusions

Aspects of the phenomenon of electroadhesion in polymer–polymer systems and some factors influencing the excitation and relaxation of electroadhesive interactions in adhesive pairs of various natures were studied.

It is shown that the basis for the formation of electroadhesive forces is the nature of dipole–dipole interactions in polymer–polymer systems. A prerequisite for good electroadhesive properties is the use of dielectric materials, with the highest values of the dielectric constant in the adhesive pair as a filling compound of electrodes used alongside a substrate, ensuring the good polarizability of electric dipoles.

With an increase in the voltage applied to the electroadhesive, the strength and range of recording the electroadhesive effect increases, and the influence of the nature of the substrate on this characteristic also increases. Materials with higher values of the dielectric constant are characterized by higher values of the electroadhesive strength of their connection and retain the ability to generate dipole–dipole interactions at greater distances.

The maximum electroadhesive forces were recorded at a gap of 0.1 mm between the plates of the electroadhesive and the substrate. It was proven that the maximum orientation of electric dipoles in the electroadhesive system is achieved at 7000 V, providing maximum electroadhesive interactions for a particular system. A further increase in the potential difference does not lead to an increase in electroadhesive interactions. It was found that the nature of the materials does not significantly affect the rate of dipole orientation under the action of an electric field—the time to reach the maximum values of the dipole–dipole interaction force for the studied materials is 6–7 s. The permittivity of the substrate material affects the kinetics of the loss of ordering of electric dipoles. The higher the values of permittivity of materials in an electroadhesive pair, the slower the misorientation of electric dipoles in the adhesive system and the longer the electroadhesive effect is preserved. An increase in the times of applying electric voltage to electroadhesive systems and relaxation after its switching off does not affect the kinetics of the subsequent charge–discharge processes of the studied systems. The activation time of electroadhesion is inversely proportional to the strength of the applied current and has a kink that determines the optimal activation time of electroadhesion, which, regardless of the nature of the studied substrates, is achieved at a current of 55 μA. With an increase in the permittivity of the materials of the adhesive system, the dependence on the coordinates activation time and current strength will be characterized by a less pronounced kink due to a decrease in the activation time of electroadhesion at low currents.

To gain a deeper understanding of the fundamental principles of dipole–dipole interactions at interphase boundaries in polymer–polymer systems, we plan to conduct further research in this area using heterogeneous phase structures of polymer systems with different surface energy characteristics, considering their polar and dispersion components.

## Data Availability

Data are available upon request to the corresponding author.
